# Aging Across the Life Course: Research Collections Available from the National Archive of Computerized Data on Aging

**DOI:** 10.1093/geroni/igab046.2472

**Published:** 2021-12-17

**Authors:** James McNally

**Affiliations:** University of Michigan, Ann Arbor, Michigan, United States

## Abstract

The creation and maintenance of sustainable data archives can be challenging, but it offers clear advantages. Properly curated data can be used by multiple researchers, testing a variety of hypotheses, and increasing the return on investment to the expensive process of data collection. Having an internally managed archival system also provides greater control and autonomy in the equitable distribution of data resources. This process ensures all researchers will have full use of the data for original research, teaching, and new directions once the data leaves the control of the local investigator’s control. This poster reviews the advantages of having a local strategy geared toward the preservation and sharing of gerontological research data. Using the National Archive of Computerized Data on Aging (NACDA) as a working example, the poster offers an overview of collections at NACDA. Using our metadata tools and variable search database, NACDA can identify studies in its collections that examine aspects of aging and health among adults during their lifecourse. Many of the studies are longitudinal or repeat measure cross-sectional studies. We are also able to identify studies that focus on aging that are not maintained by NACDA but which are available to interested researchers. Using a strategy of archival preservation combined with a strong focus on productive research Innovation in Aging, NACDA has amassed data and metadata covering a wide array of studies worldwide that address the aging lifecourse. Because our collections are multinational, we share these data at no cost to interested users worldwide

